# A Mobile App for Hypertension Management Based on Clinical Practice Guidelines: Development and Deployment

**DOI:** 10.2196/mhealth.4966

**Published:** 2016-02-02

**Authors:** Hannah Kang, Hyeoun-Ae Park

**Affiliations:** ^1^ Systems Biomedical Informatics Research Center College of Nursing Seoul National University Seoul Republic Of Korea

**Keywords:** mobile health, clinical practice guideline, hypertension, self-management

## Abstract

**Background:**

Hypertension is a chronic and lifestyle-related disease that requires continuous preventive care. Although there are many evidence-based clinical practice guidelines (CPGs) for hypertension management, applying them to daily management can be difficult for patients with hypertension. A mobile app, based on CPGs, could help patients with hypertension manage their disease.

**Objective:**

To develop a mobile app for hypertension management based on CPGs and evaluate its effectiveness in patients with hypertension with respect to perceived usefulness, user satisfaction, and medication adherence.

**Methods:**

The hypertension management app (HMA) was developed according to the Web-Roadmap methodology, which includes planning, analysis, design, implementation, and evaluation phases. The HMA was provided to individuals (N=38) with hypertension. Medication adherence was measured before and after using the HMA for 4 weeks. The perceived usefulness and user satisfaction were surveyed in the patients who completed the medication adherence survey.

**Results:**

Of the 38 study participants, 29 (76%) participated in medical adherence assessment. Medication adherence, as measured by the Modified Morisky Scale, was significantly improved in these patients after they had used the HMA (*P*=.001). The perceived usefulness score was 3.7 out of 5. The user satisfaction scores, with respect to using the HMA for blood pressure recording, medication recording, data sending, alerting, recommending, and educating about medication were 4.3, 3.8, 3.1, 3.2, 3.4, and 3.8 out of 5, respectively, in the 19 patients.

**Conclusions:**

This study showed that a mobile app for hypertension management based on CPGs is effective at improving medication adherence.

##  Introduction

Hypertension is a disease that can lead to myocardial infarction, cerebral infarction, and heart failure [1], and its prevalence rate is 29.2% among males and 24.8% among females [2]. Controlling blood pressure is essential in hypertension care.

The two main branches of hypertension care are lifestyle improvement and drug treatment. According to the 2014 Evidence-Based Guideline for the Management of High Blood Pressure in Adults of the Eighth Joint National Committee [1], lifestyle management is the first step in hypertension care for patients older than 18 years. The second step is setting a blood pressure target based on the patient’s age, presence of diabetes mellitus, and chronic renal disease status, and reaching that target through drug treatment. This indicates the importance of helping patients with hypertension to maintain lifestyle improvements and drug treatments in order to keep their blood pressure in check by fostering self-management skills.

The use of mobile health care is becoming increasingly popular in the self-care of chronic diseases such as hypertension. Mobile health care refers to the delivery of health care services via mobile communication devices [3]. The widespread availability of mobile phones with app capabilities means that they can be used to facilitate mobile health care via various types of interventions. According to an analysis of the current state of mobile health care, mobile phones can now be used to provide health care intervention strategies, such as tracking patient data, providing tailored self-management, leveraging social influence, and utilizing entertainment [4].

This expansion of mobile health care led to approximately 20,000 commercial health care apps appearing on the iPhone App Store in 2013 [5]. The main functions of most hypertension management apps are measuring blood pressure and managing records. Providing users with more clinically helpful functions requires the use of evidence-based knowledge when developing apps. For this purpose, a paper-based clinical practice guideline (CPG) needs to be converted into a computer-interpretable guideline and applied to the delivery system. In other words, hypertension management, through mobile health care, requires the development of an app that provides tailored information and recommendations on lifestyle management based on accurate evidence while also improving the hypertension medication adherence of the user. The development and correct utilization of such an app could help patients with hypertension improve their lifestyle and increase their medication adherence through drug education and medication reminders.

The aim of this study is to develop and evaluate a mobile app for hypertension management based on CPG knowledge, and then apply the developed hypertension management app (HMA) to patients with hypertension and evaluate its effects with respect to perceived usefulness, user satisfaction, and medication adherence.

## Methods

The HMA was first developed based on CPG knowledge, and evaluated by experts. The HMA was then applied to patients with hypertension to investigate the perceived usefulness and user satisfaction, while its effect on medication adherence was evaluated using a one-group pre- and posttest design.

### Development of the HMA

The HMA was developed using the planning, analysis, design, implementation, and evaluation phases of the Web-Roadmap methodology of information science [6].

#### Phase I: Planning

The CPGs for providing the evidence for hypertension management were selected in consultation with experts whilst also considering the credibility of the publisher, year of publication, and inclusion criteria of the information. After selecting each CPG, knowledge was extracted from it that would improve the adherence to treatment for hypertension management.

**Figure 1:**
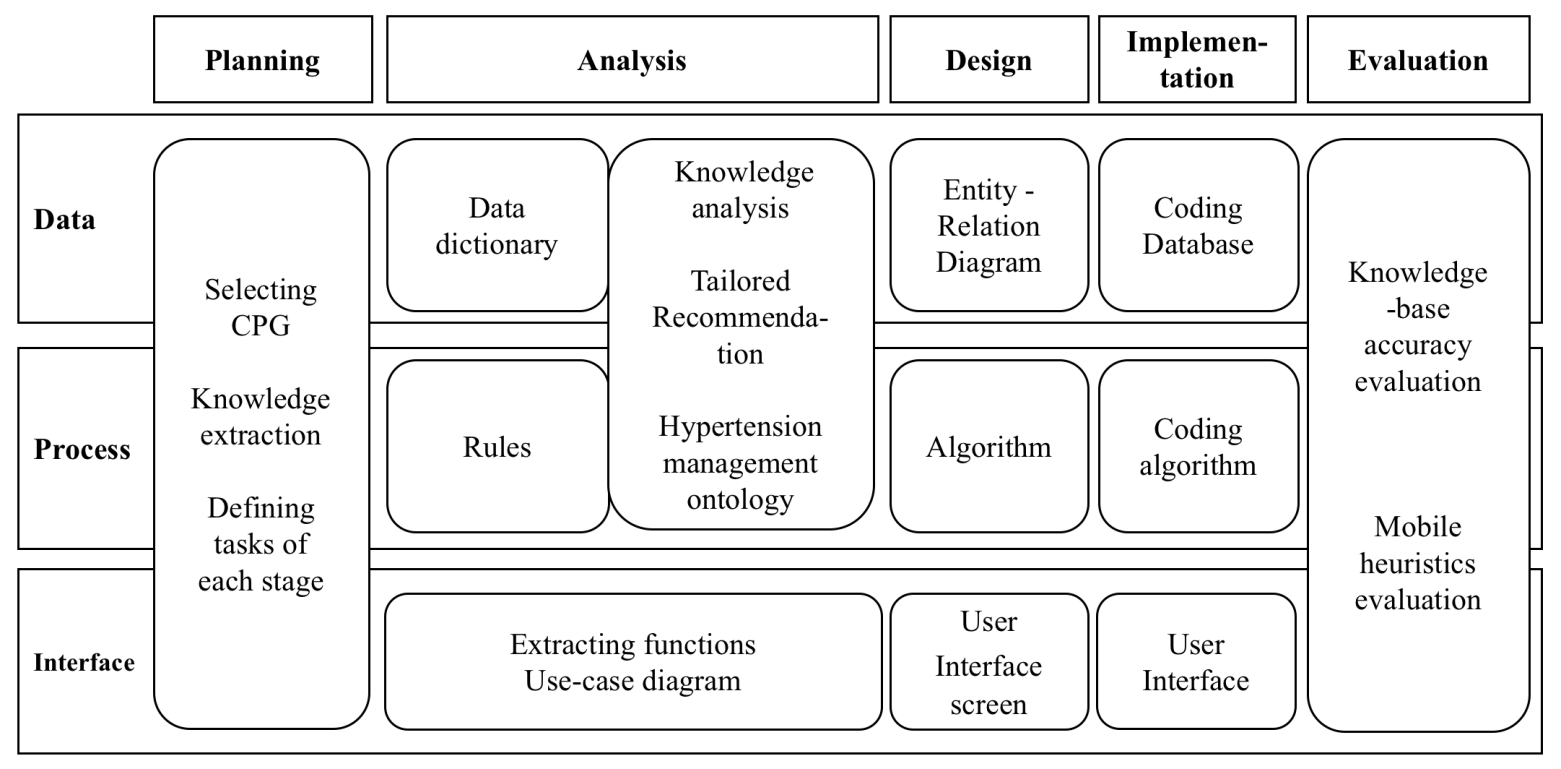
Development stages of the HMA.

The Web-Roadmap methodology of information science [6] was used as a reference to define the data, process, and interface domains of the planning, analysis, design, and implementation phases, which in turn were used to plan the tasks and products of each phase (Figure 1).

#### Phase II: Analysis

In the data domain, the data necessary for the system were extracted from the CPG and grouped. Data dictionaries were developed for each item.

In the process domain, rules to serve as judgment criteria for each intervention were identified and summarized. These rules were later used for algorithm development. In addition, extracted data and rules were organized into the hypertension management ontology to apply the content of hypertension management according to the nursing process. Data and rules were also used to compile a list of tailored recommendations to make to patients with hypertension.

In the interface domain, the required functions of the HMA were extracted by analyzing the requirements of the HMA. The requirements for the user interface were presented in a use-case diagram.

#### Phase III: Design

In the data domain, an entity-relation diagram was created to design a database for the data extracted in the analysis phase. Data entities of the system were identified and presented in tables with their attributes then connected with lines based on their relationships. This procedure revealed the data organization of the system.

In the process domain, knowledge extracted in the analysis phase was elaborated in algorithmic form for use in system operations. In the interface domain, the layout of the user interface was designed to implement essential functions extracted in the analysis phase.

#### Phase IV: Implementation

In the implementation phase, the database, algorithms, and user interface were realized through coding.

#### Phase V: Evaluation

The HMA was first evaluated on the accuracy of the knowledge base and then mobile heuristics. The system development stage was completed by reflecting the evaluations of experts in revising the app.

##### Accuracy of the Knowledge Base

**Figure 2:**
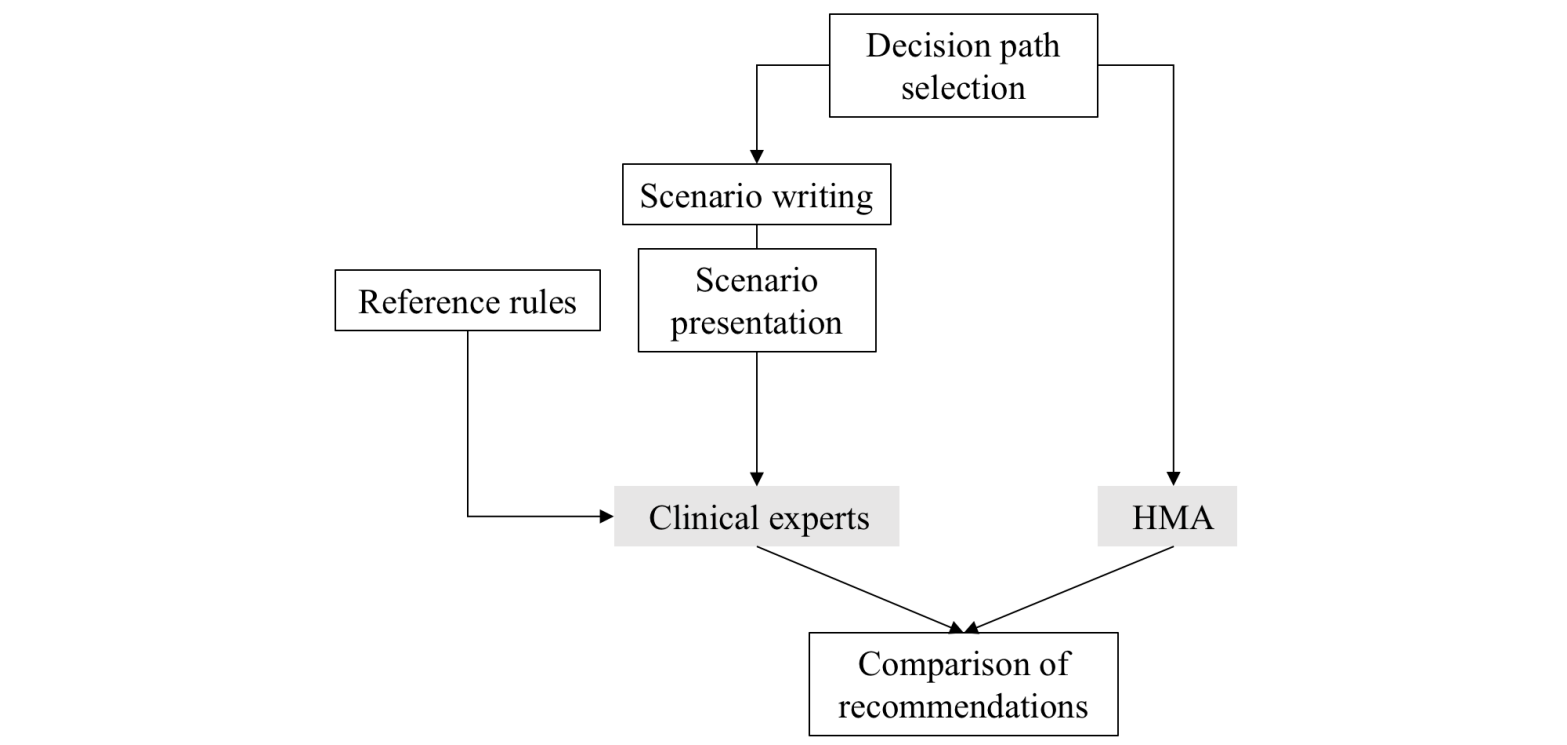
Process used to evaluate the accuracy of the knowledge base.

The degree of consistency between recommendations provided by experts and by the HMA was evaluated based on a previous study [7]. This evaluation was performed by 3 nurses, holding doctor of philosophy degrees (PhDs) in adult health nursing, and who have a special interest in chronic diseases. The accuracy of the knowledge base was evaluated using the process depicted in Figure 2.

Seven scenarios were created to ensure that all possible judgments in decision-making nodes appeared at least once. Knowledge extracted from the CPG was documented as reference rules and provided to experts. Scenarios were presented to the evaluators, who were then asked to write recommendations in a free-text format. The same data were entered into the system, and the recommendations produced by the system were compared with those of the clinical experts and checked for inconsistencies. If inconsistencies between the recommendations or their details were found, the opinions of the experts were used to improve the system.

##### Mobile Heuristics Evaluation

The expert evaluations of the usability of the HMA were performed by applying mobile heuristics evaluation. In accordance with Nielsen’s recommendation to involve 3-5 experts [8], 5 evaluators took part in the mobile heuristics evaluation. The evaluators were experts with at least a PhD in nursing informatics and medical informatics, and with prior experience in medical system development. The mobile heuristics was evaluated using the 8 principles of mobile heuristics [9], which is a modification of the 10 principles of heuristics evaluation [8] modified appropriately for assessing mobile software. Each evaluation item was scored on a severity ranking scale to diagnose the number and distribution of flaws in the software and the opinions of the evaluators. The Korean version of the tool was used for this study [10]. The eight principles of mobile heuristics evaluation were introduced to the evaluators, who were provided with Android-based mobile phones that included the HMA. After using the HMA, the experts were asked to freely comment on any issues about usability pertaining to items on the heuristics, and evaluate the severity of each problem on a scale from 0 to 4. Items were indicated as faulty if 2 or more people scored them as 1 point or higher on the severity ranking scale. Any items that received 4 points were considered to have major usability problems and required revision. The system was revised for these items.

### Deployment of the HMA

#### Subjects

The minimum number of subjects for this study was found to be 38 based on the requisite for a *t* test with an effect size of 0.55, alpha of .05, and a power level of .95, as proposed in a previous study [11]. All calculations were performed using the software G*Power. Patients with hypertension visiting cardiovascular clinics at tertiary hospitals in Seoul, South Korea who were taking 1 or more antihypertensive drug(s) were recruited in person. Those who possessed a mobile phone, were able to use a mobile app without outside help, understood the purpose of this study, and volunteered their participation were chosen as subjects.

#### Procedure

The study participants were briefed on its purpose and their initial medication adherence was examined in person. The researcher installed the HMA on the participant's mobile phone, and then taught the participant how to use it. The study participants were then asked to use the HMA for 4 weeks. The perceived usefulness, user satisfaction, and final medication adherence were then examined via a phone call or in a face-to-face interview. The participants kept following their treatment plans as advised by health care providers during the study period.

#### Measurement Scales

##### Perceived Usefulness

The perceived usefulness of the HMA was evaluated using 6 questions pertaining to perceived usefulness from Davis’ perceived usefulness and perceived ease-of-use measurement scales [12]. Cronbach alpha for the 6 perceived usefulness items was .98. There were significant correlations of .63 and .85 between perceived usefulness and self- reported current usage and self-predicted future usage, respectively [12]. The Korean version of the tool [13] was used. Perceived usefulness was measured on a scale from 1 (“strongly disagree”) to 5 (“strongly agree”). The final score for usefulness was defined as the mean of the scores for all of the questions.

##### User Satisfaction

User satisfaction was evaluated with respect to using the HMA for blood pressure recording, medication recording, data sending, alerting, recommending, and educating about medication, on a scale from 1 (“very dissatisfied”) to 5 (“very satisfied”). Cronbach alpha for the 6 user satisfaction items in this study was .78. The score for each question was defined as the user satisfaction for the relevant function.

##### Medication Adherence

Medication adherence was measured using the Modified Morisky Scale (MMS) [14]. MMS has two factors: motivation subscale items (factor 1), and knowledge subscale items (factor 2). Cronbach alphas for the two factors were .722 and .691, respectively, in a previous study [15]. The scale is composed of 6 questions using a yes-or-no survey, with 0 or 1 point assigned to each choice. The final MMS score was the sum of the answers to these 6 questions.

#### Statistical Analysis

The obtained data was analyzed using Microsoft Excel and SPSS. Subjects’ demographics and hypertension characteristics were analyzed using real numbers, percentages, means, and standard deviations, while the perceived usefulness, user satisfaction, and MMS scores were analyzed using means and standard deviations. The one-group pre- and posttest MMS scores for medication adherence were measured via a non-parametric Wilcoxon signed-rank test.

#### Ethics and Informed Consent

This study was approved by Samsung Medical Center Institutional Review Board (IRB No. 2014-03-130-005), where the participants were recruited. The informed consents contained the purpose, procedure, and measurement scales of this study. They were obtained at cardiovascular clinics of Samsung Medical Center in Seoul, South Korea, by paper. For safety and security reasons, personal identifiers were encoded and collected data were kept in a secured storage location to restrict access.

## Results

### Development of the HMA

#### Phase I: Planning

##### Selection of CPGs

The hypertension CPGs were selected by searching well-known CPG search websites such as the National Guideline Clearinghouse [16], the Guidelines International Network[17], and the National Institute for Health and Care Excellence [18], using the following keywords: “hypertension management,” “hypertension,” “hypertension treatment,” and “high blood pressure.” Consulting with an expert group majoring in nursing informatics and considering the publisher’s credibility, year of publication, and inclusion criteria, 5 CPGs were selected (Table 1).

##### Extraction of Hypertension Management Interventions

Of the 5 CPGs, 4 (80%) included a hypertension diagnosis by the physicians and principles of anti-hypertensive drug prescriptions. These were omitted from the present study because they were not under the purview of the HMA. What interventions the HMA could provide in terms of nursing and self-management were used to extract intervention categories and items for hypertension management (Table 2) and the target systolic blood pressure for hypertension treatment (Table 3).

**Table 1:** CPGs selected for the HMA.

CPG	Publisher	Country	Date	Reference
2014 Evidence-based guideline for the management of high blood pressure in adults	American Medical Association, The Eighth Joint National Committee	United States	2014	[1]
2013 Hypertension guideline of the Korea Society of Hypertension	The Korea Society of Hypertension	Republic of Korea	2013	[19]
European Society of Hypertension (ESH) and European Society of Cardiology (ESC) guidelines for the management of arterial hypertension	European Society of Hypertension and European Society of Cardiology	Members of the European Union	2013	[20]
The clinical management of primary hypertension in adults	National Institute for Health and Care Excellence (former National Institute for Health and Clinical Excellence)	United Kingdom	2011	[21]
Nursing management of hypertension	Registered Nurses’ Association of Ontario	Canada	2005	[22]

**Table 2:** Intervention categories and targets [20,22].

Category		Target
**Lifestyle intervention**		
	Diet	Sodium intake of 65-100 mmol/day
	Body weight	Body mass index(BMI) <25 kg/m^2^
		Waist circumference <102 cm (men)
		Waist circumference <88 cm (women)
	Exercise	Dynamic exercise (more than moderate effort) for >30–60 min 4-7 times/week
	Alcohol	Maximum of 2 standard drinks/day or 14 standard drinks/week (men)
		Maximum of 1 standard drink/day or 9 standard drinks/week (women)
	Smoking	Smoking cessation
	Stress	Susceptibility scale score <30^a^
Medications		Obtaining users’ medication history
		Providing education about medications that are prescribed for users
Monitoring and follow-up		Receiving appropriate follow-up
Documentation		Documenting and sharing information with the user and health care team

^a^To learn how to cope with stress effectively.

**Table 3:** Target systolic blood pressure [1].

Characteristics of the patient	Target systolic blood pressure
Age <60 years	140 mmHg
Age ≥60 years with underlying disease^a^	140 mmHg
Age ≥60 years without underlying disease	150 mmHg

^a^Diabetes mellitus and/or chronic renal disease.

#### Phase II: Analysis

##### Data Domain

A total of 41 data items were extracted for hypertension management in the planning phase. The extracted data were categorized into two groups: input data and calculated data. Data dictionaries of each data item were developed by specifying the type, values, and unit.

To facilitate obtaining data on sodium intake and stress level from the users, 2 questionnaires that were not part of the CPGs were used as replacements for the criteria listed in Table 2. For sodium intake, a diet behavior questionnaire [23], with 12 items, was developed in order to classify groups with high and low sodium intakes. Whereas for stress level, the Brief Encounter Psychosocial Instrument, Korean version (BEPSI-K) scale [24], containing 5 items, was used.

##### Process Domain

For the process domain, rules were extracted from the CPG to provide tailored recommendations for each participant. For setting the targets for each hypertension management item according to the participant's demographic data, 8 rules were extracted (Table 4), and 20 rules were extracted for tailored recommendations based on preset target and input data.

##### Hypertension Management Ontology and Tailored Recommendation List

**Figure 3:**
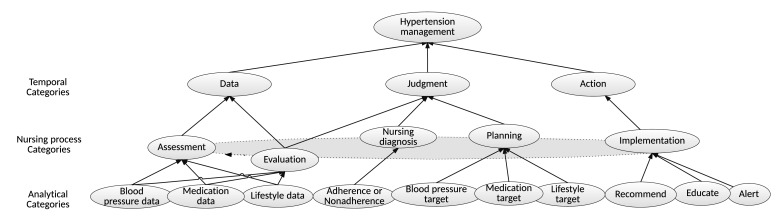
Hypertension management ontology of the HMA.

To allow the hypertension management flow of the HMA to be visualized by linking concepts with the nursing process, an ontology for hypertension management (Figure 3) was developed based on a previous study [25]. The ontology is made up of three levels where concepts of the analytical level are linked to the nursing-process-categories level according to their roles in the HMA. Nursing process categories are linked to data, judgment, and action concepts, which constitute the hypertension management concept of the HMA.

A tailored recommendations list was developed based on CPG knowledge by combining data extracted from the data domain and rules extracted from the process domain. This process produced 17 tailored recommendations including one maintenance recommendation and one modification recommendation each for the 7 target items of blood pressure, sodium intake, exercise, alcohol, smoking, body weight, and waist circumference, as well as 3 recommendations for low, moderate, and high stress levels. In addition, optional detailed recommendations were made for subgroups of each recommendation when necessary. Each tailored recommendation comprises the following 3 parts: (1) informing the target of the item, (2) providing the evaluation result, and (3) providing the recommendation. The evaluation-result sentence provides the result of input data analysis, and the recommendation sentence provides evidence and recommendations from the clinical guidelines.

##### Interface Domain

**Figure 4:**
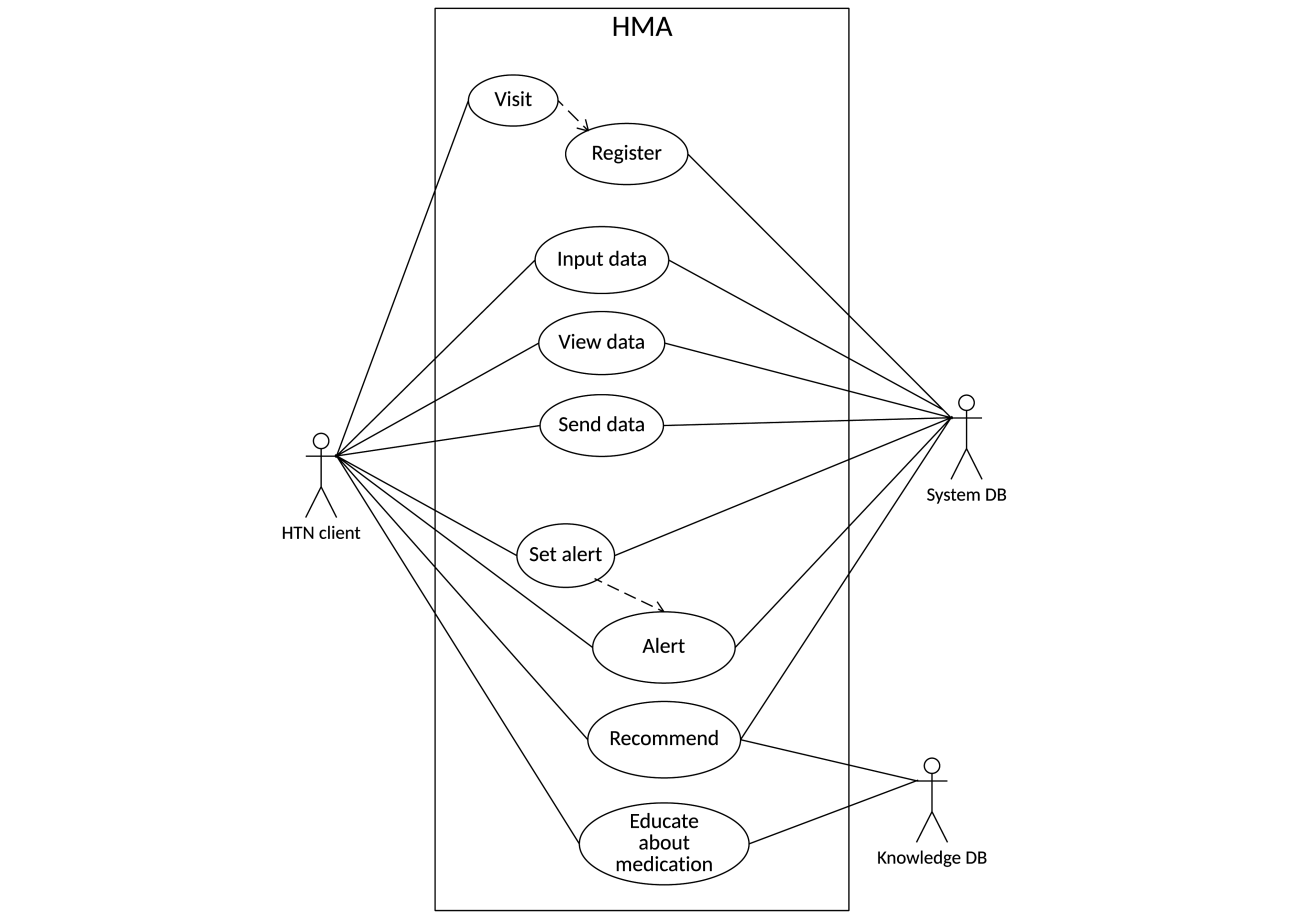
Use-case diagram of the HMA.

Forming an expert group and extracting the functions necessary for the HMA yielded 9 functions, which are presented in a use-case diagram (Figure 4) and are described in Textbox 1.

The use-case diagram shows the actors, use cases, and their relationship in the HMA. The 3 actors outside of the system are the system database, the knowledge database, and user. The system contains 9 use cases that are connected to actors according to their interactions.

**Description of HMA function.**

1. Visit: visit from the system
2. Register: register the user to the system
3. Input data: input user’s initial, medication, blood pressure, and lifestyle data
4. View data: view patient’s saved data as a chart, calendar, and graph
5. Send data: send patient’s saved data via e-mail
6. Set alert: set an alert for a medication time or hospital visit date
7. Alert: alert about a medication time or hospital visit date
8. Recommend: give user targets and recommendations
9. Educate: provide education about the medication

**Table 4:** Rules of setting targets of the HMA [20,22].

Target category	Rules for set targets
Blood pressure	If ≥60 years with no underlying disease, then systolic BP <150 mmHg
	Else systolic BP <140 mmHg
Sodium intake	Diet behavior score <5
Body weight	Body weight (kg) <25 × height^2^ (m^2^)
Alcohol	If male and weight ≥60 kg, then alcohol intake ≤2 glasses/day and ≤14 glasses/week
	Else alcohol intake ≤1 glasses/day and ≤9 glasses/week
Smoking	<1 cigarette/day
Stress	Brief Encounter Psychosocial Instrument, Korean version score ≤1.6
Waist circumference	If male, then waist circumference <102 cm
	Else waist circumference <88 cm
Exercise	Exercise frequency >4 times/week, exercise duration >60 min, exercise intensity >moderate effort

#### Phase III: Design

##### Data Domain

**Figure 5:**
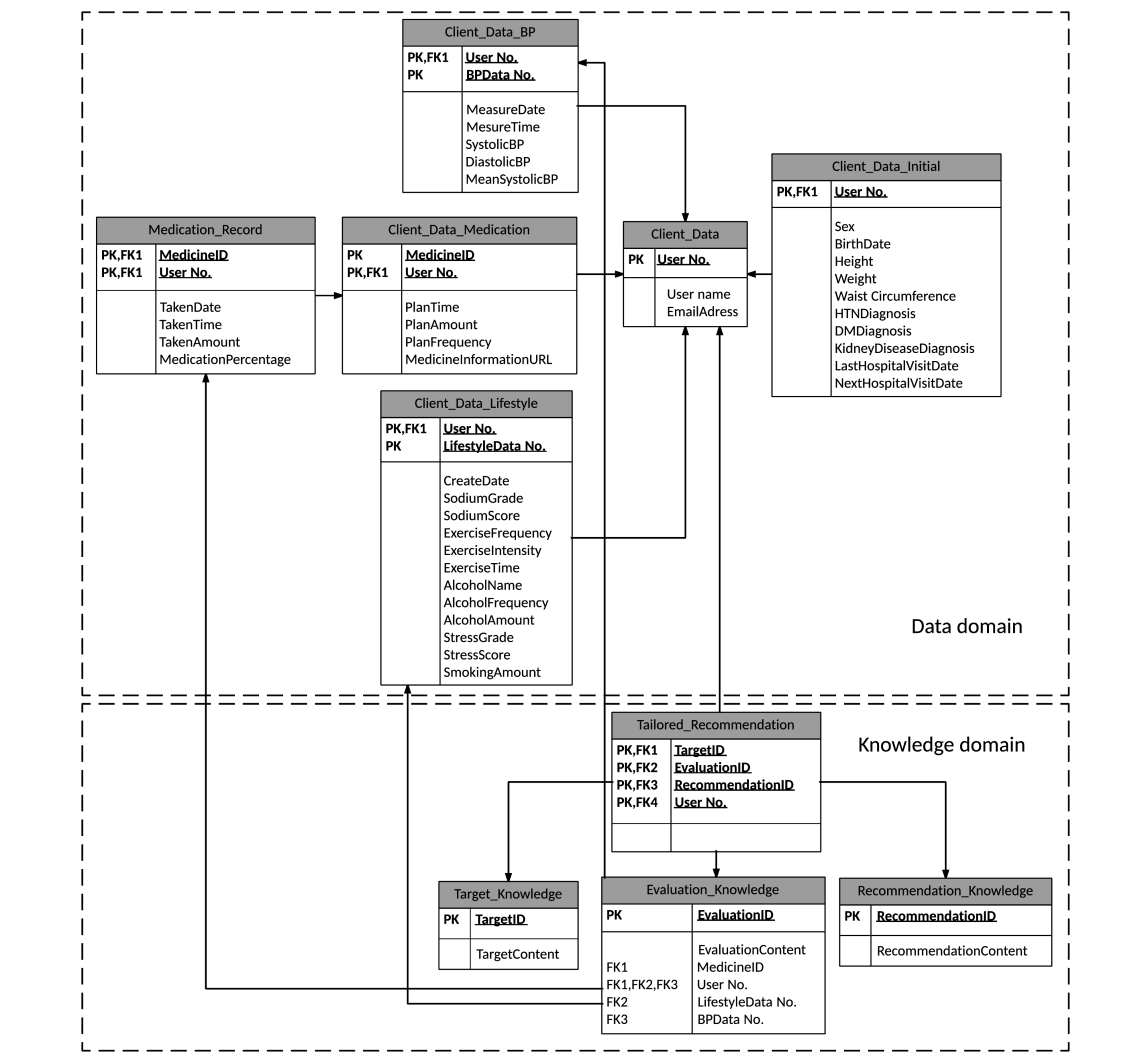
Entity-relation diagram of the HMA.

The data structure is documented in an entity-relation diagram (Figure 5). Separate tables were prepared for the data and knowledge domains, and the data domain tables were further divided according to the change cycle of data values.

##### Process Domain

**Figure 6:**
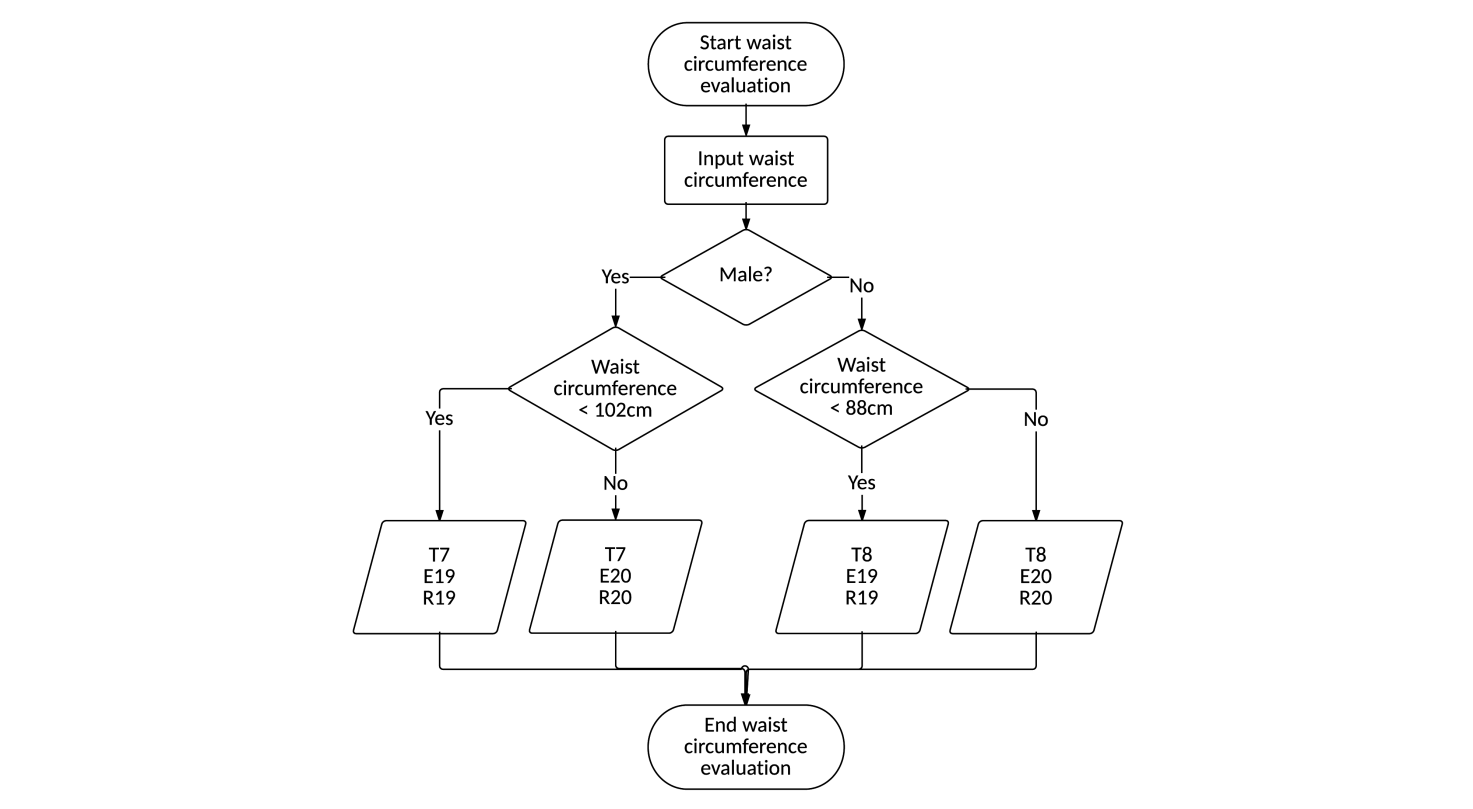
Algorithm for waist-circumference evaluation.

By linking the 8 rules for setting the target and the 20 rules for tailored recommendations, 8 algorithms were developed. The algorithms were used to set targets for each intervention item and provide tailored recommendations based on the target and input data. As an example, the algorithm for evaluating the waist circumference is shown in Figure 6. When a user inputs his/her waist circumference, depending on the user’s sex and target waist-circumference evaluation (T7 or T8), the evaluation result (E19 or E20) and recommendation (R19 or R20) will be presented to the user.

In the case of a female user whose waist circumference is 92 cm, a recommendation composed of T8 (“Your target waist circumference is 88 cm”), E20 (“Your waist circumference is 92 cm, 4 cm over the target”), and R20 (“For a hypertension patient, maintaining the proper waist circumference is necessary to lower blood pressure. Reduce your waist circumference to 88 cm”) will be received.

##### Interface Domain

The user interface screens were designed around a menu containing essential functions extracted in the analysis phase. In total, 34 user interface screens were designed.

#### Phase IV: Implementation

##### System Environment

The HMA was developed using the Android SDK Platform 4.4.2 Java Development Kit and Eclipse. The database management program was developed using MYSQL. The system works on mobile phones running the Android operating system versions 2.3 to 4.4.

##### System UI Implementation

The HMA developed in this study was implemented with 5 basic menus at its core (Textbox 2).

**The five basic menus of the HMA.**

**Figure 7:**
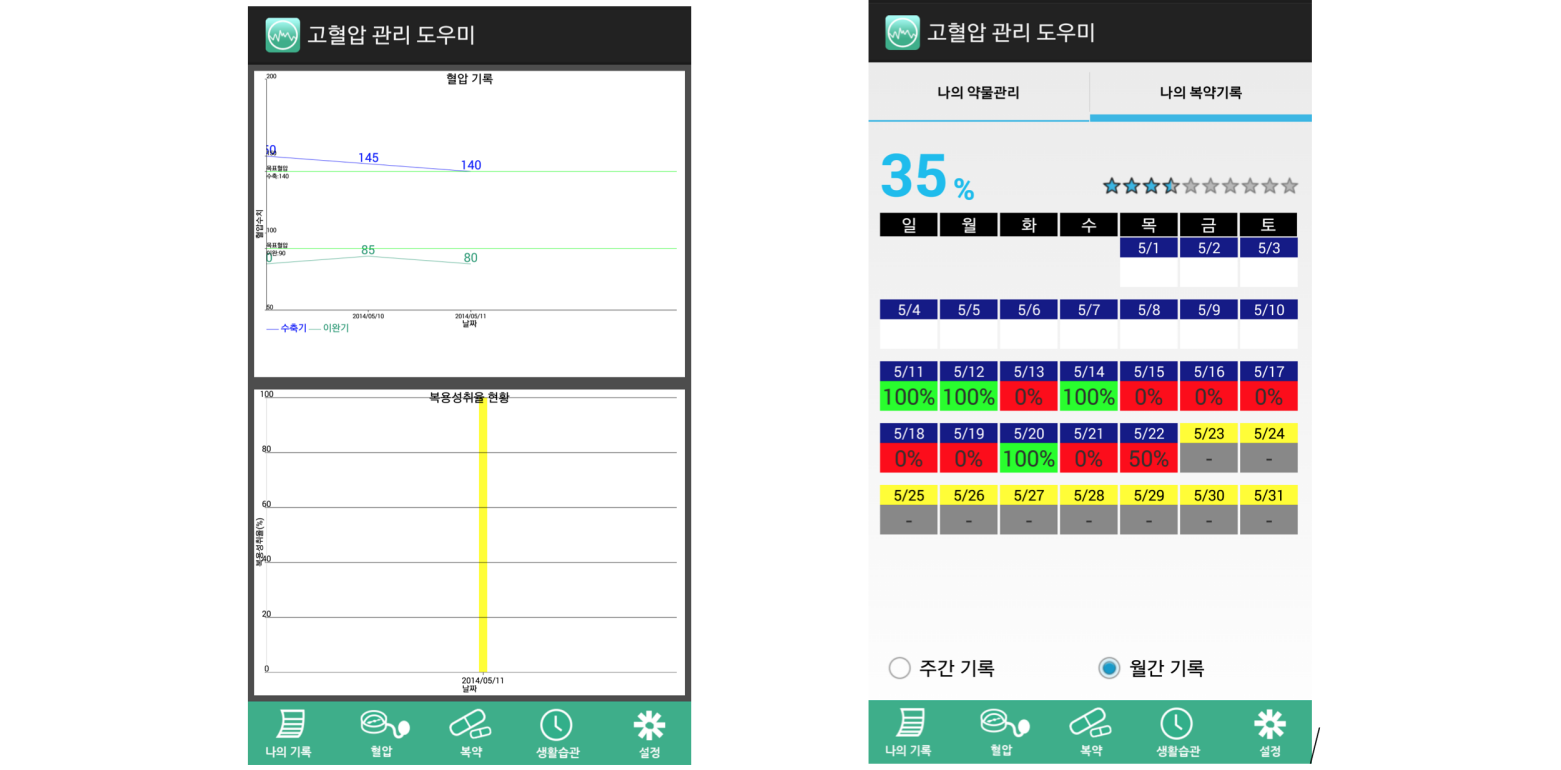
User interfaces of the "my records" (left) and "medication management" (right) menus.

1. *My records*, which shows the most recent blood pressure and hypertension medication usage records in a graph (Figure 7).
2. *Blood pressure management*, which can be used to check the target blood pressure, makes input blood pressure measurements, receive tailored blood pressure management recommendations, and review blood pressure records.
3. *Medication management*, which can be used to browse for the user’s hypertension medication information, enter the medication schedule to be provided with reminders, record the taking of medication, and review the adherence on a weekly and monthly basis (Figure 7).
4. *Lifestyle management*, which provides tailored recommendations in response to the user’s answers to questions regarding the 7-lifestyle management items (ie, sodium intake, body weight, waist circumference, exercise, alcohol, smoking, and stress). This menu displays a pop-up window once a month to alert the patients to input lifestyle data.
5. *Settings*, whose basic functions include information management, alert settings, password settings, and data sending via e-mail.

#### Phase V: Evaluation

##### Knowledge-Base Accuracy Evaluation

In this evaluation, 2 assistant professors teaching adult health nursing and 1 senior nursing researcher participated. All 3 evaluators had more than 10 years of clinical practice experience. There were no inconsistencies in the broader recommendations produced by the evaluators and the HMA. However, the evaluators proposed the following three additional detailed recommendations: (1) adding the option to input the daily amount of alcohol intake in addition to the existing weekly amount of intake, (2) more specific comments on sodium intake, and (3) adding “get advice from a professional” to stress management. These three recommendations were added to the HMA.

##### Mobile Heuristics Evaluation

The mobile heuristics was evaluated by 5 nursing informatics, medical informatics, and computer programming experts with PhDs. They flagged 33 usability-related problems related to the mobile heuristics items, of which 3 problems that were related to a severity score of 4, and 7 problems that were flagged by more than 2 evaluators were revised after reflecting on the evaluators’ comments.

### Deployment of the HMA

#### Demographics of Study Subjects

Initially 38 study participants were recruited, of which 29 (76%) participated in the medication adherence survey and 19 (50%) participated in the perceived usefulness and user satisfaction surveys. The mean age of the subjects was 56.0 years, with the highest percentage (48%, 14/29) of them being in the age group of 50-59 years. Most (66%, 19/29) of the participants were male, 62% (18/29) were educated to higher than a bachelor’s degree, 62 % (18/29) were employed full-time, 62% (18/29) were religious, 93% (27/29) were married, and 72% (21/29) were living with their spouse and children.

#### Hypertension-Related Characteristics of Study Participants

Hypertension-related characteristics of the study participants are presented in Table 5. A mean of 106.2 months had passed since the participants were diagnosed with hypertension, and 101.2 months since they had started taking hypertension medication. Most of the participants (79%, 23/29) reported taking a hypertension medication, and 90% (26/29) took medication once a day. Of the participants, only 5 (17%, 5/29) experienced side effects of hypertension drugs, 55% (16/29) had a family history of hypertension, and 66% (19/29) had previously received education about hypertension. The two main sources of hypertension information were hospitals (52%, 15/29) followed by the Internet (28%, 8/29).

**Table 5:** Hypertension-related characteristics of the study participants (N=29).

Characteristic	Number or response	n (%)
Number of antihypertensive drug taken per day	1	23 (79)
	2	2 (7)
	3	4 (14)
Medication frequency per day	1	26 (90)
	2	3 (10)
Number of pills taken per day	1	23 (79)
	2	2 (7)
	3	4 (14)
Experience of side effects	Yes	5 (17)
	No	24 (83)
Prior education about hypertension	Yes	19 (66)
	No	10 (34)
Source of hypertension information	Hospital	15 (52)
	Community health center	1 (3)
	TV, radio, newspaper	4 (14)
	Internet	8 (28)
	Book	2 (7)
	Other sources	3 (10)
	Non-response	2 (7)

#### Survey Results

##### Perceived Usefulness

The overall mean perceived usefulness score was 3.7 out of 5. The items with the highest score (3.9) and lowest score (3.4) were “I would find the HMA useful in my hypertension management” and “Using the HMA in my hypertension management would increase my productivity,” respectively. The mean score for perceived usefulness of each item is listed in Table 6.

##### User Satisfaction

The mean user satisfaction scores for 6 functions were (1) 4.3 for blood pressure recording; (2) 3.8 for medication recording; (3) 3.1 for data sending; (4) 3.2 for alerting; (5) 3.4 for recommending; and (6) 3.8 for educating about medication.

##### Medication Adherence

The mean (SD) MMS scores increased significantly (*P*=.001) between before and after using the HMA, from 4.2 (1.3) to 5.2 (1.1), respectively.

**Table 6:** Perceived usefulness score by item.

Number	Item	Mean score
1	Using the HMA in my hypertension management would enable me to accomplish tasks more quickly	3.5
2	Using the HMA would improve my hypertension management performance	3.8
3	Using the HMA in my hypertension management would increase my productivity	3.4
4	Using the HMA would enhance my effectiveness in hypertension management	3.8
5	Using the HMA would make it easier to manage my hypertension	3.8
6	I would find the HMA useful in my hypertension management	3.9

## Discussion

### Development of the HMA

In the knowledge extraction phase, it was necessary to decide from which CPG, and to what extent knowledge needs to be extracted, in order to form the knowledge base for the HMA. After selecting multiple CPGs, knowledge on hypertension diagnosis and drug therapy required for health care providers to make decisions was excluded. Instead, the following knowledge relevant to a patient’s self-management of hypertension was included: target blood pressure, lifestyle management intervention, and evidence for tailored recommendations.

The system development stage involved converting the knowledge extracted from the CPG into a computer-interpretable form so that it could be applied to the system. Data and rules were extracted from the text-formatted CPG, then coded so that both the user and the system could understand them. The process of making the CPG knowledge understandable to computers requires cooperation between clinical experts and computer developers [26]. This process would be more efficient if a knowledge modeling tool and system development methodology, specialized for the development of a CPG-based mobile system, are developed.

The evaluation of the knowledge-base accuracy indicated that the recommendations proposed by the experts were more detailed than those suggested by the HMA. Since the accuracy of the knowledge base was evaluated after the HMA had been developed, coding needed to be performed twice to reflect this change. This extra coding step could be avoided if the expert evaluation of the knowledge base was performed in the algorithm development stage.

In the mobile heuristics evaluation, diverse expert opinions were sought from evaluators with diverse backgrounds, such as nursing, medical informatics, and computer programming. The various evaluators made different numbers of comments on different heuristics items. Since the capability of each evaluator acts as an important variable in mobile heuristics evaluation [27], including diverse experts from several fields facilitated the identification of usability problems.

### Deployment of the HMA

The users found the HMA useful. According to Davis [12], there is a positive correlation between system acceptance and perceived usefulness. Although system acceptance was not directly measured in the present study, we assume that users will have a positive attitude toward using an app for measuring hypertension.

Users rated that they were highly satisfied with the HMA’s blood pressure and medication recording functions. This is in accordance with a previous study on a chronic disease management mobile app [28], which found patients suffering from chronic diseases to be very interested in its recording functions. Recording functions are especially useful for those who need to record and monitor their health information on a chronic basis.

The education provided about medicine information also received a high score of 3.8 in the user satisfaction survey, which is attributed to the HMA being connected to an external database and provides the latest information on hypertension medicine in real-time, including visual information. The quality of information is an important factor in determining the success of information systems [29]. This is consistent with the present study where users were satisfied with the well-constructed knowledge base because it provided quality information on medicine.

On the other hand, users showed a lower user satisfaction for the data sending function. The purpose of this function was not only to store, but also to send the users’ records to the users’ health care providers for feedback. However, because the HMA manages the users’ blood pressure records, provides tailored recommendations, and offers analysis of their medications, the users may have not found it necessary to send their records to their health care provider, resulting in a lower score for the data sending function. The HMA was rated as useful but inefficient for hypertension care in the survey of the perceived usefulness. The users were satisfied with the monitoring functions themselves (eg, blood pressure and medication recording), but rated the efficiency aspect of the HMA poorly due to the additional act of recording, since the users previously did not have to do this.

This study found that the HMA improved medication adherence. However, an attrition bias [30] may have been present since 9 of the 38 participants (24%) dropped out during the 4-week study period. Those who answered the posttest survey may have represented a high-adherence group, and thus not representative of the entire survey cohort. Results that are more accurate are likely to be obtained in a future study that includes both control and experimental groups with similar initial adherence rates.

### Limitations

Implications of the study findings are limited, as we did not measure the direct effects of the HMA on parameters such as actual medication adherence, lifestyle improvement, and change in blood pressure. A future study should measure the actual medication adherence using digital adherence measuring equipment [31], and evaluate lifestyle improvements and blood pressure changes over the long term. A future app with wireless medical sensors and technologies for better data collection and transmission will allow this future study possible.

There is the possibility that those with low medication adherence refused to participate in the survey. Thus, it is necessary to explore the reasons for the withdrawals to evaluate selection bias on the medical adherence survey. In addition, it is arguable that the 4-week intervention was too short and the number of participants involved was not sufficient to measure the effectiveness of the HMA. This will certainly introduce bias in the measurements. A future study with a longer intervention period and a larger study cohort is recommended.

Finally, we did not assess patient engagement and use of the HMA for their hypertension management, which should be taken into consideration in future studies.

### Conclusions

This study describes the development of a mobile HMA based on CPGs that allows patients with hypertension to conduct appropriate self-management. CPGs were used as the knowledge base of the HMA, and critical functions were constructed to aid 2 factors of hypertension management: lifestyle improvement and drug treatment. The developed HMA was evaluated based on the accuracy of the HMA’s knowledge base and its mobile heuristics, was applied to a cohort of patients with hypertension patients, and evaluated for its perceived usefulness and user satisfaction. The HMA scored 3.7 out of 5 for perceived usefulness, and with respect to user satisfaction, it scored 4.3 for blood pressure recording, 3.8 for medication recording, 3.1 for data sending, 3.2 for alerting, 3.4 for recommending, and 3.8 for educating about medication. The findings also demonstrated that medication adherence increased significantly after using the HMA (*P*=.001).

This study is significant not only in that it developed a mobile app for hypertension management based on CPGs, but also evaluated the developed system and its effects on patients with hypertension over a 4-week period. Further studies are necessary to determine the direct effects of the HMA on patients with respect to actual medication adherence, lifestyle improvement, and change in blood pressure.

## Abbreviations

BEPSI-K: Brief Encounter Psychosocial Instrument, Korean version
BMI: body mass index
CPG: clinical practice guideline
HMA: hypertension management app
MMS: Modified Morisky Scale
